# Exploration in the Presence of Mother in Typically and Non-typically Developing Pre-walking Human Infants

**DOI:** 10.3389/fnbeh.2020.580972

**Published:** 2020-11-13

**Authors:** Tzviel Frostig, Hanna Alonim, Giora Scheingesicht, Yoav Benjamini, Ilan Golani

**Affiliations:** ^1^Department of Statistic and Operations Research, School of Mathematical Sciences, Tel Aviv University, Tel Aviv, Israel; ^2^The Mifne Center, Rosh Pinna, Israel; ^3^Bar Ilan University, School of Social Work, Ramat-Gan, Israel; ^4^The Sagol School of Neuroscience, Tel Aviv University, Tel Aviv, Israel; ^5^School of Zoology, Faculty of Life Sciences, Tel Aviv University, Tel Aviv, Israel

**Keywords:** behavioral homology, ASD, autism, mother related exploration, homebase, ASD prodrome, excursions

## Abstract

In previous phenotyping studies of mouse and rat exploratory behavior we developed a computational exploratory data analysis methodology including videotaping, tracking, preparatory methods for customized data analysis, a methodology for improving the replicability of results across laboratories, and algorithmic design for exposing the natural reference places (origins) used by animals during exploration. We then measured the animals’ paths in reference to these origins, revealing robust, highly replicable modules termed excursions, which are performed from the origin into the environment and back to the origin. Origin-related exploration has been claimed to be phylogenetically conserved across the vertebrates. In the current study we use the same methodology to examine whether origin-related exploration has also been conserved in human pre-walking typically developing (TD) and a group of non-typically developing (NTD) infants in the presence of their stationary mother. The NTDs had been referred to a center for the early treatment of autism in infancy by pediatric neurologists and clinicians. The TDs established a reference place (origin) at mother’s place and exhibited a modular partitioning of their path into excursions performed in reference to mother, visiting her often, and reaching closely. In contrast, the NTDs did not establish a distinct origin at the mother’s place, or any other place, and did not partition the exploratory path into excursions. Once this difference is validated, the differences between the human infant groups may serve as an early referral tool for child development specialists. The absence of distinct modularity in human infants at risk of autism spectrum disorder can guide the search for animal models for this disorder in translational research.

## Introduction

A conspicuous spatial regularity in the exploratory behavior of many organisms is that of a reference place in relation to which they explore the environment. In the wild, many animal species have a home site to which they return regularly after exploring their home range or territory, be they, for example, wolves (Fritts and Mech, 1981), small mammals (Brown, 1966), ants (Martin and Rudiger, 1988) bumble bees (Woodgate et al., 2016), or millipedes (Hoffmann, 1984). In behavioral neuroscience experiments, rats have been shown to explore the experimental arena from a reference place, from which they perform excursions into the environment (Eilam and Golani, 1989). The high accumulation of time spent across a large number of visits also characterizes the reference place of, for example, mice (Fonio et al., 2009), zebra fish (Stewart et al., 2010, 2011), and infant rats (Loewen et al., 2005). This reference place, often termed a “home base,” exerts its influence on the organism’s behavior across the entire exploratory basin. Visits to the home base partition the path into separate excursions in the environment. The latter are further partitioned into progression segments and staying-in-place (lingering) episodes (Drai and Golani, 2001). In moment-to-moment behavior in a novel environment the excursions grow in extent (Benjamini et al., 2011) and differentiate from simple excursions to complex ones (Benjamini et al., 2011).

The performance of exploratory excursions has also been reported in the wild in infant primates – rhesus monkeys (Berman, 1980), baboons (Altmann and Samuels, 1992), and chimpanzees (van de Rijt-Plooij and Plooij, 1987). As the infants of these primates develop, they perform increasingly longer excursions from mother into the environment and back to mother. While the mother is often on the move during the performance of such excursions, the excursions nevertheless involve both exploration and active management of distance in reference to an origin or base by the infant, be it a mobile or stationary mother.

Using an arsenal of computational and statistical tools, we set out to study whether the establishment of an origin at mother’s place, and a modular organization of behavior in reference to this origin indeed applies to human pre-walking infants exploration of a novel environment (with the mother stationary in it); whether this architecture also applies to non-typically developing (NTD) infants; and whether mother-related exploration is homologous to origin-related exploration performed in reference to, e.g., home base behavior in vertebrates.

In the current study we compared the behavior of five TD human infants to that of seven NTD infants. The latter were referred to the Mifne Center for the early treatment of autism in infancy (Alonim, 2004): Four infants were referred for developmental assessment by expert clinicians and three were referred for treatment by pediatric neurologists. All the NTDs had primary assessment due to parental concerns regarding their infants’ development. For six of these seven infants we have current information (at least 2 years after the session): four are in special education schools and 2 are in mainstream schools following one or 2 years of intensive therapy. The parents of the five TDs were similarly contacted, and reported to have no developmental problems in recent years, and all TDs attend mainstream schools. For more details about NTDs see Supplementary Material.

**FIGURE 1:**
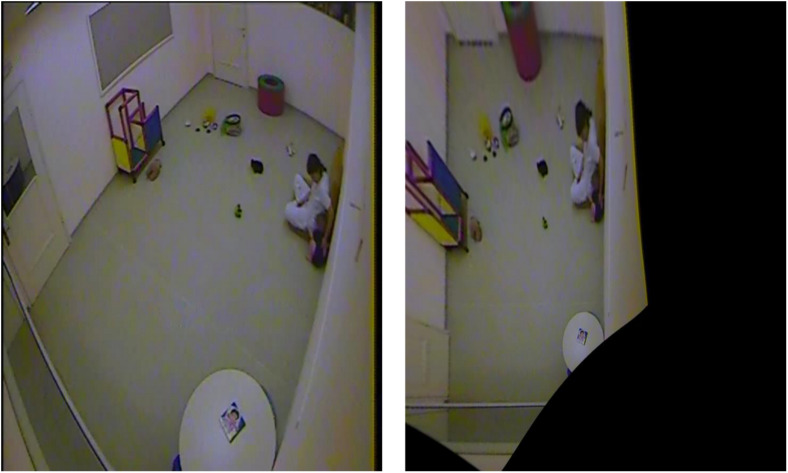
Mifne Treatment room. Before **(left)** and after transformations **(right)**. Note the furniture, toys, mother’s location (here with infant sitting on her lap), on the right side near the wall, and two doors (on the left – exit doorway and on top door leading to bathroom).

All infants were recruited at the pre-walking stage of stable crawling, and each participated with the respective mother in a video-taped half-hour session conducted in a medium-sized room. In each trial the mother entered the room carrying her infant, sat on a mattress near the wall, and then seated the infant or let him or her slide down next to her. The mother was requested to remain seated and allow the infant to act freely; and mothers complied. Two video cameras were used throughout the session: one capturing a view of the whole room including the infant and the mother (Figure 1); and another zooming in on the infant and following it. The behavior of the infant was video-taped and then tracked (see section “Materials and Methods”). In the analysis, we explicitly ignored the infants’ or the mothers’ behavior at the scale of segmental articulations, facial expressions, and vocalizations, focusing on path structure of the infant.

In our preliminary observations of the path properties we were impressed by the contrast between the strong modularity and partitioning of the TD infants path-session into excursions from the mother and back to her, and the paucity or even absence of excursions in the behavior of the NTD infants. Having studied and reviewed exploratory behavior in a wide range of species and situations (Golani and Benjamini, 2018) we have not encountered so far a similar deficit and contrast. The wide taxonomic distribution of origin-related exploration, furthermore, marks it as a candidate behavioral homology. We thus tuned our efforts to establish the following two hypotheses.

Our first hypothesis in this exploratory study is that mother-related exploration in TD infants is partitioned into excursions performed in reference to an origin established at mother’s place. The quantitative properties of this behavior can be measured and analyzed, supporting our theory that this structure of exploration is homologous across species and situations. Such evidence would increase the translational value of measures compared across human infants and prospective animal models.

Our second hypothesis is that NTDs exhibit a deficit in the establishment of an origin at mother’s place, and in the partitioning of exploration into mother-related excursions. Once supported by quantitative evidence, this deficit can be used as an early warning signal for child development specialists. The novel algorithms designed by us to examine these hypotheses are necessary for understanding the organization of exploratory behavior and are part of the results of this study.

Plotting the path traced by infants in the presence of a stationary (Hoch et al., 2018), or a moving (Thurman and Corbetta, 2017) caregiver has been performed previously, aiming at various functional aspects, but ignoring the modular partitioning of behavior into excursions. One study, plotting the path traced by 5.8 ± 3.1 years old children in two 3 min sessions revealed that those on the autism spectrum tended to remain at a distance from their parents, exhibiting a longer latency to approach them (Cohen et al., 2014).

The presence of mother-related exploration across the primates, and the structural architecture it shares with other instances of origin-related exploration (Golani and Benjamini, 2018), prompts us to also examine its translational status as a behavioral homology across the vertebrates. Our results provide not only a glance into a conserved, relatively universal structure regardless of the specific functions it fulfills in human infants, but also a glimpse into the distinct operational worlds of the TDs and a sub group of NTDs, and into the ways in which the infants attend to the world and come to grips with it.

## Materials and Methods

Subjects were twelve 8–18 months-old pre-walking infants recruited to participate in a videotaped half hour session conducted at the Mifne Center. All infants were boys except for one NTD girl (Adva). All infants were documented at the stage of stable crawling. Using an age criterion for this type of exploratory study was problematic because infants reach a stable crawling stage at a somewhat variable age. This also reduced the concern for equal crawling proficiency across groups [but note the claim that autism does involve motor and locomotor deficiencies and abnormalities which are of its essence (Teitelbaum et al., 1998; Torres et al., 2013; Buja et al., 2018)]. Five were TD infants whose parents had volunteered to participate. The TD infant parents had not raised any concerns regarding developmental problems. The research procedures, the methods of analysis, the storage of data and the use of fictitious names were approved by the Ethics Committee of Tel Aviv University. The parents were asked to sign a form of consent, consisting of their informed consent, agreeing to participate in the experiment and allowing use of the data collected. The form included a description of the research and was approved by the Ethics Committee of Tel Aviv University. Informed consent for the publication of Figure 1 was obtained from the mother and father of the infant presented in that figure. All methods were performed in accordance with the relevant guidelines and regulations. The NTD infants underwent external expert developmental assessments, including the verification of inclusion and exclusion, detailed family history, perinatal, medical, and developmental history, and physical and neurological checkups. (While diagnosis at this early age has been controversial, recently issued NIH RFAs “encourage research that would develop and validate new screening methods for autism spectrum disorders (ASD) that can be used in infancy at 0–12 months of age”: grants.nih.gov/grants/guide/rfa-files/RFA-MH-19-120.html: R01; grants.nih.gov/grants/guide/rfa-files/RFA-MH-19-121.html: R21).

The mother entered the trial room carrying her infant, sat on a mattress in the periphery of the room, and then seated the infant next to her. The mother was requested to remain seated within the area of the mattress and allow the infant to act freely for a 30-min session. Some sessions were stopped earlier if the infant appeared to be distressed, but all were filmed and tracked for at least 20 min.

Common to all NTD mothers had been a concern about their infants’ health, so it could be argued that the poor modularity exhibited by their infants is a property of the NTD mother-infant interaction. We are, however, prevented from describing the observed paucity of excursions as an interactional property. Because we intentionally minimized maternal responsiveness, our assessment was not dyadic, and our study is descriptive, having no pretense to causal mechanisms.

A video camera capturing a static view of the whole room including the infant and the mother (Figure 1), and another zooming in on the infant and following it, were used. The static camera was situated at a high side view angle, so the 2D coordinates in the video image (Figure 1, left) were transformed to a top view (Figure 1, right), using a projective transformation (implemented in Matlab) which was calculated from 4–5 points whose coordinates were known for both bases. Data were prepared for segmentation (Drai et al., 2000; Drai and Golani, 2001; Hen et al., 2004) and were analyzed by SEE, a publicly available Software for the Exploration of Exploration developed and elaborated by us over the course of many years^1^ (Drai and Golani, 2001). *Room***:** The infants were tracked in a medium-sized, 3.65 m by 5.45 m room. *Tracking*: The tracking of the infant was done manually using a specifically dedicated program in Matlab. The infant’s location was tracked every ∼15 frames; missing coordinates were completed using linear interpolation. Since asking mothers to remain passive and tracking them would have been counterproductive we refrained from tracking mother’s (stationary) location. One feature of the current study was a focus on the structure at the path scale, neither the infants’ nor the mothers’ movements of the parts of the body, vocalizations, etc., were tracked.

We resorted to the use of manual tracking after exhausting other possibilities: different tracking algorithms had failed due to bad image quality, multiple object moving and the fact that an infant is a large object, so even when tracking was successful, there was jitter across the infant’s body.

### The Ring Algorithm for Defining a Visit to a Place

A ring is centered at the place, with inner circle of radius *r*_*in*_ and outer circle of radius *r*_out_ > *r*_in_. A visit at the place starts by entering the inner circle and ends by leaving the outer circle. Since visits at places are often associated with lingering, this algorithm screens re-entrances resulting from noisy behavior within the ring. The radia *r*_out_*andr*_in_ were varied to ensure results comparing number of visits defined this way do not rely critically on the particular pair of values used.

### Cumulative Dwell-Time Maps (Heat Maps)

The construction of the heat maps involved several steps:

(1) Obtaining the original coordinate data from the Matlab tracking.
(2) Smoothing the coordinates using the SEE program (see footnote text 1).
(3) Dividing the room into a grid, in which each cell is a 1(cm)^∗^1(cm), and calculating time spent in each cell according to 2.
(4) Smoothing the cells using 2D Gaussian smoother: calculating for each cell a new value according to the weighted average of the cell itself and its neighboring cells. The weights are given by a 2D Gaussian kernel withσ = 14 (truncated 31 cm away).
(5) Finding local maxima of the smoothed dwell time and discarding the 96% of maxima’s with lower values.
(6) Calculating the number of visits to each local maxima, using the ring algorithm with (*r*_in_ = 30, *r*_out_ = 50).

### Physical Proximity of Centers of Mass Plots

The proximity plot exhibits the timing, duration, and extent of the infant’s being in proximity with mother and with furniture and doors in the room. Each set of concentric circles exhibits the behavior of a specific infant. Starting at twelve o’clock and proceeding clockwise for the session’s duration, the arc traced on the circle’s circumference and the colored section of the circle, designate the time of start, the time of end, the duration in session percentage, and the extent of the entry into mother’s or any other large object’s close proximity (the infant seemingly casts a shadow on the peripheral area separated from the circle by its entry). The color of the polygon stands for the visited object, with mother being colored in black. Furniture items are designated by specific colors. For radius *R*, time *t* and for object *j* a point is drawn according to *I*(*D*_j,t_ < *R*), *D*_*j,t*_ being the distance of the infant at time *t* from the center of object *j*. In order to compare multiple pieces of furniture of different sizes, the distance drawn on the plot are the distance of the infant from the object minus the radius of the object. See Supplementary Figure S1.

### Segmentation to Excursions

To partition the infant’s path across the session into excursions (forays away and back to the mother, roundtrips), we needed to define for each mother a customized circumscribed place she occupied. The length of the radius tracing the boundary of mother’s place in reference to mother’s center influenced the number of visits paid by the infant to mother across the session: the smaller the radius the fewer the number of visits. To obtain a customized place around the location defining mother’s center, we used the ring algorithm to define the visits varying *r*_*in*_ from 30 to 120 cm from the mother’s center of mass, and keeping*r*_out_ = 1.1*r*_in_. Counting the number of visits paid to mother for each *r*_in_, the longest radii interval in which there was no change in the number of visits served as an indication for a region of stability. The smallest radius of that interval was used as *r*_in_ of the ring that defines a visit at the mother, and thereby allows the segmentation into excursions, and the calculation of the number of visits paid to mother and thereby also the number of excursions performed from her into the environment.

### Statistical Testing

All testing results are reported by observed *p*-value. We used the term statistical significance when the *p*-value was ≤ 0.05, but further adjusted for multiplicity using the Benjamini–Hochberg (BH) procedure (Benjamini and Hochberg, 1995) controlling the false discovery rate at 0.05. For the comparison of endpoints and for comparisons of the number of excursions per minute to mother (see Table 1), the Wilcoxon rank sum test was used. The effect size is measured as $\frac{\left|\bar{x}-\bar{y}\right|}{s_{\text{p}}}$, where *X* represents the measures of the TD group, *Y* the measure of the NTD group. $s_{\text{p}}^{2}$ is the pooled variance, $s_{\text{p}}^{2}=\frac{\left(n_{\text{x}}-1\right)\,s_{\text{x}}^{2}+\left(n_{\text{y}}-1\right)\,s_{\text{y}}^{2}}{n_{\text{x}}+n_{\text{y}}-2}.$ For these, some of the original measures were transformed as detailed in Table 1, to increase the symmetry of the distributions and allow informative visualizations (note that such monotone transformations do not affect the Wilcoxon rank sum test results). For all tests *n*_x_ = 5, *n*_y_ = 7. For comparison of the dwell time with the mother and the number of excursions from the mother per minute as a function of the defining distance of mother’s proximity, permutation test was used (see Supplementary Table S1). The test utilizes the fact that under the null hypothesis there should be no difference between the TD infants and NTD infants’ curves.

**TABLE 1:** Summary of statistical comparisons between the TD and NTD infants.

Wilcoxon rank sum test	*P*-value	Adjusted *p*-value (BH)	Means Difference $\bar{x}-\bar{y}$	Statistic (W)	SD (pooled)	Effect size $\frac{\left|\bar{x}-\bar{y}\right|}{S\,D}$
Sqrt (# of excursions to mother per minute) (Infant’s distance to mother closer than 100cm)	0.00252	0.0088	0.23	35	0.080	2.93
Logit proportion of time near mother (Infant’s distance to mother closer than 100cm)	0.00252	0.0088	1.98	35	0.987	2.01
Average progression speed	0.14899	0.1490	4.98	27	6.167	0.80
Logit proportion of room covered	0.04798	0.0560	0.77	30	0.494	1.57
Average speed outside mother vicinity (Infant distance to mother larger than 100cm)	0.01010	0.0177	3.12	33	1.687	2.07
Sqrt (# of contact episodes per minute)	0.03030	0.0424	0.30	31	0.186	1.60
Logit proportion of contact time	0.00505	0.0118	2.40	34	1.161	2.06

*It should be noted that the comparison between the number of excursions per minute and proportion of dwell time is dependent on the radius that defines the mother’s position. The difference remains robust to changes in that radius (see Supplementary Figure S3).*

## Results

### Dwell Time Distribution Across the Room

**FIGURE 2:**
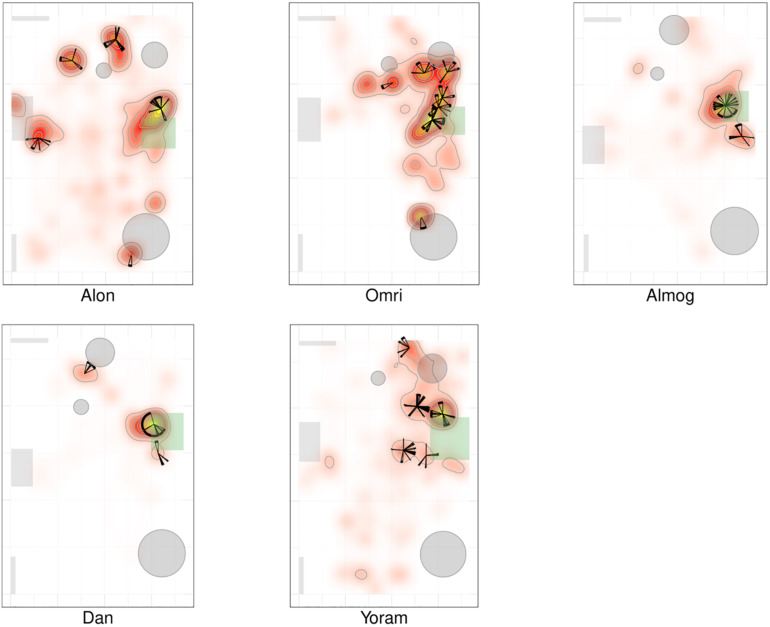
Spatio-temporal summary of location for typically developing (TD) Infants. The TDs most preferred place was located near mother and they visited it regularly paying it the highest number of visits. The figure presents the smoothed cumulative dwell-time spent in different locations across the observation room, and the number of visits paid by the infants to neighborhood locations exhibiting peak dwell-times. Number of visits is obtained using the two concentric circles method (*r*_in_ =  30cm, *r*_out_ = 50cm, see section “Materials and Methods”). Dwell-time is represented by colored contour lines forming a topographical map. The contour lines are spaced at the quantiles (0.1, 0.2, 0.3,…) of the smoothed cumulative dwell-time (see section “Materials and Methods”). Note that in these heat maps the color is proportional to the dwell time of each specific infant. Spokes wedges and stars respectively stand for single visits, visit durations, total number of visits to that place, and relative timing starting at twelve o’clock, all extended over the normalized session time (see Figures 5, 6).

**FIGURE 3:**
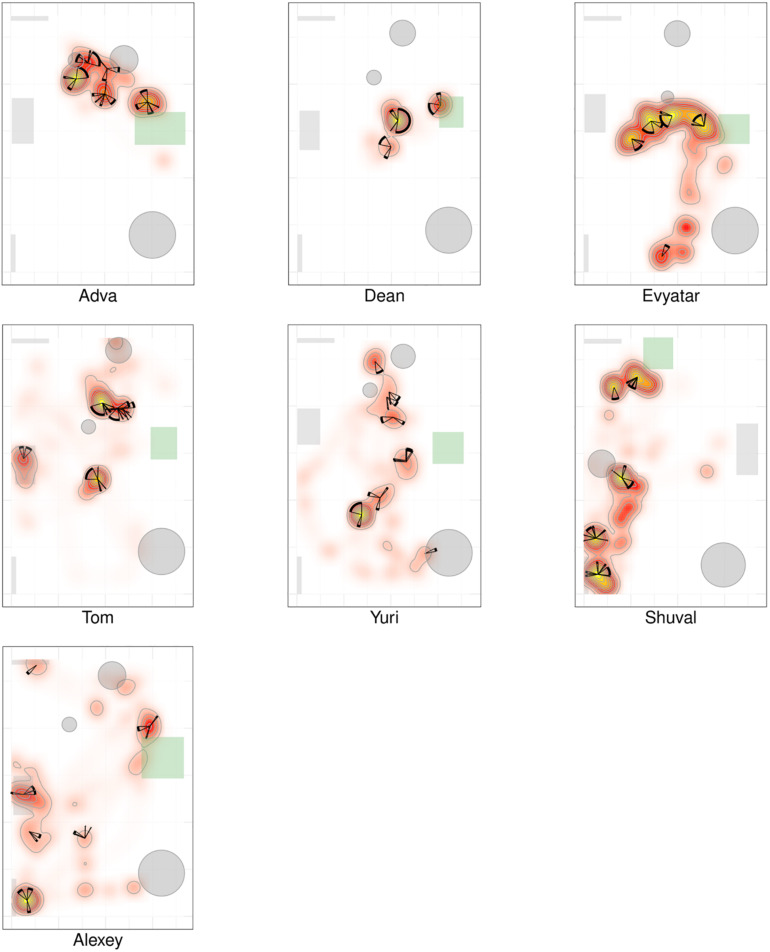
Spatio-temporal summary of location for non-typically developing (NTD) Infants. The NTDs’ most preferred places were not located near mother and the infants did not visit these places regularly, nor paying to them the highest number of visits. Furthermore, none of the NTDs established any other location marked by both highest cumulative dwell time and highest number of visits. For further explanation see Figure 2.

Figures 2, 3 present a visual representation of the smoothed cumulative dwell-time that both the TD and NTD infants spent in different locations across the observation room and the number of visits paid by them to those neighborhood locations exhibiting peak dwell times (see section “Materials and Methods”). Dwell-time is represented by colored contour lines forming a topographical map. For example, comparing the TD infant Alon with the NTD infant Tom (all names provided in this study are fictitious), reveals that dwell time displays a patchy distribution across the room in both infants. However, whereas for Alon (TD) there was a single peak, located near mother, which stood out in terms of dwell-time (signified by a yellow center), for the NTD infant Tom there were two peaks of relatively the same dwell-time and both were located away from mother. Alon (TD) spent time over the whole room whereas Tom (NTD) adhered to the upper half, hardly lingering in the lower half of the room. Similar differences were revealed for most of the infants in the respective groups: all the TDs exhibited a single preferred place in terms of dwell-time, located near mother, whereas the NTDs tended to exhibit more than one preferred dwell-time place, located away from mother.

Whereas the TD infants tended to establish preferred places also near furniture, the NTDs often established places away from both walls and furniture. Most of the NTDs established peak dwell-time places in the open space with their bodies facing their mother yet always keeping a distance from her. Two NTDs (Shuval and Alexey), established peak dwell times *vis-à-vis* the door leading out of the room.

While the TDs tended to cover the whole room, the NTDs tended to adhere to only part of the room (see section “Endpoint Summaries” – proportion of room covered). TD median coverage 0.042 vs. NTD median coverage of 0.017 (see Table 1).

#### Visit Distribution Across High Dwell-Time Places

Figures 2, 3 provide a spatio-temporal summary of the process by which dwell time was *allocated* across the session to the main places. The stars on top of the peak places represent the number, order, and duration of the visits (see section “Materials and Methods”) to the respective peak dwell-time places. Starting at 12 o’clock, the circumference of the stars represents the session’s normalized length. Proceeding clockwise, each wedge presents a visit to this location, with its start and end times given by the orientation, and its duration thus presented by the arc’s length. For very short visits the wedge appears as a single spoke. Almog (TD), for example, visited the most preferred place, located by his mother, 14 times, thus paying the highest number of visits to the place near mother and accumulating time near mother in a piecemeal manner, whereas Dean (NTD) visited his most preferred place only 3 times, ignoring that place for a third of the session and visiting mother only twice. These differences in visit management apply between most of the two groups of infants: all TDs most visited place was near mother for the highest number of times; whereas all the NTDs’ most visited places were located away from mother and each was typically ignored for substantial parts of the session. In summary, the NTDs did not show sustained attention to any place, or object as expressed in spread-across-the-session visits. In both groups visits to most places (except for the TDs mothers’ places) were sparse and not evenly distributed. Both Alexey and Shuval (NTDs) paid the highest number of visits to the exit doorway.

It should be noted that the number of visits to peak dwell-time places does not exhaust the number of visits to mother, and therefore does not disclose the full number of excursions performed from the mother; these visits merely refer to one or at most two places in her vicinity. The infants might, and indeed did, visit mother’s vicinity from other, less visited directions, not necessarily belonging to the peak dwell time places.

The observations of (i) the stable preference of the TDs for the place located near the mother, (ii) the absence of a preferred place near the NTDs’ mother (Figures 2, 3), and (iii) the absence of any other preferred place (marked by both highest dwell time and highest number of visits across the session) implied that the NTDs did not establish any other origin, nor any other origin-related behavior. This justified an examination of all the infants’ path-sessions in explicit reference to mother’s location (see section “Endpoint Summaries” – # of excursions per minute to mother and proportion of time spent near mother).

### The Itinerary, Duration, and Extent of Physical Proximity Between the Centers of Mass of the Infant and the Mother During Visits to Mother

**FIGURE 4:**
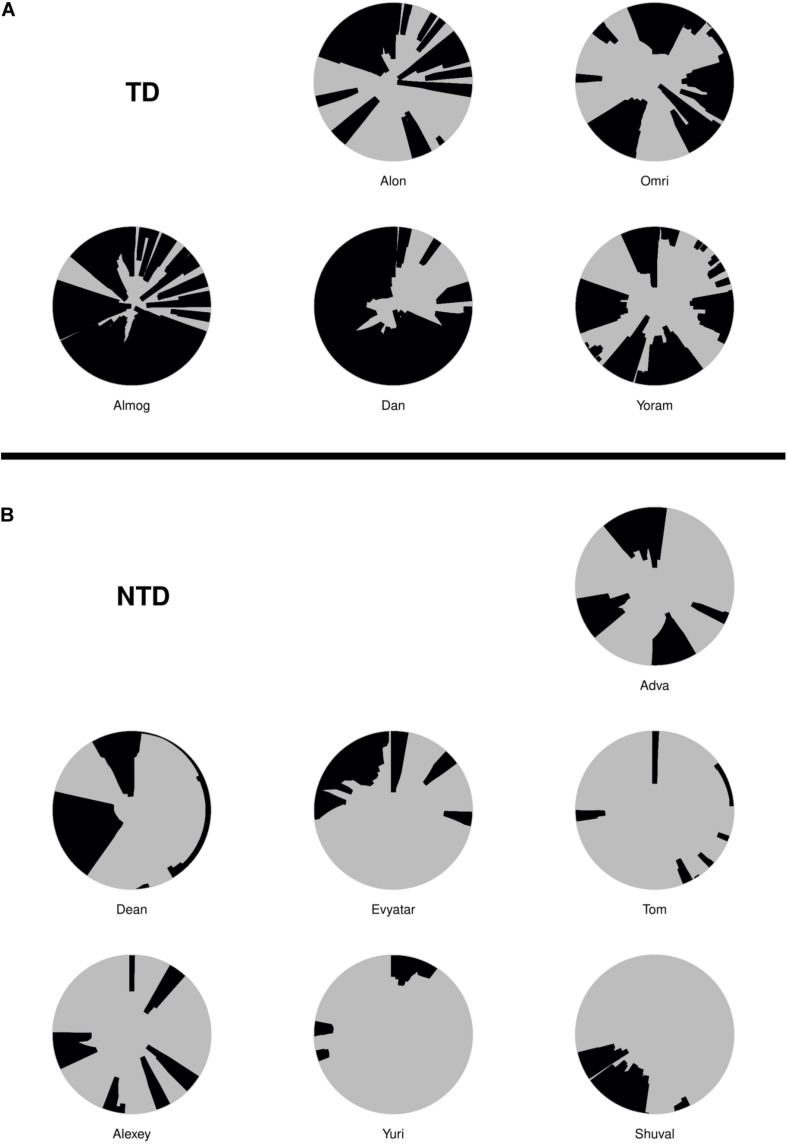
Management of time and distance from mother. The typically developing (TDs; A) visit mother frequently and persistently, exhibiting close physical proximity of their two centers of mass and for long durations, whereas the non-typically developing (NTDs; B) visit mother rarely; and when visiting they typically merely approach and do not touch mother. The physical proximity of centers plots exhibit the timing, duration, and extent of the infant’s being in close proximity with mother. The concentric circles are centered on mother’s location, spanning a radius of 120 cm around the center. Starting at twelve o’clock and proceeding clockwise for the session’s duration, the arc traced on the circle’s circumference and the colored section of the circle designate the times of start, end, and duration, and the extent of physical proximity to mother (see section “Materials and Methods”).

For the duration of the entire session, we plotted in the correct order, the relative time of start and end, the duration in session percentages, and the extent of the infant’s proximity to mother; noting whether the infant merely approached the mother, had direct contact, and/or climbed on mother (see section “Materials and Methods”). As shown, Alon (TD, Figure 4a) visited mother’s place 12 times, sometimes only approaching her and at other times climbing on her; whereas Tom (NTD, Figure 4b) spent the first few seconds at the very start of the session near mother and then later slightly approached her three times. More generally, the TDs came to close, extended, and persistent grips with mother, whereas all the NTDs tended to avoid mother’s proximity for extended parts of the session. Adva and Alexey, two NTDs, did visit mother across the session, but for a relatively small number of visits, without penetrating deeply into the space she occupied. Note that in many infants the last extended visit involved climbing mother, yet it has been absent in four of the NTDs. The difference in the extent of physical proximity to mother is also evident in the plots presented in Supplementary Figure S3 of both the average proportion of time spent, and the number of visits paid at distances starting from zero centimeters from the center of her position (Supplementary Figure S3).

### The Infants’ Management of Distance From Mother

**FIGURE 5:**
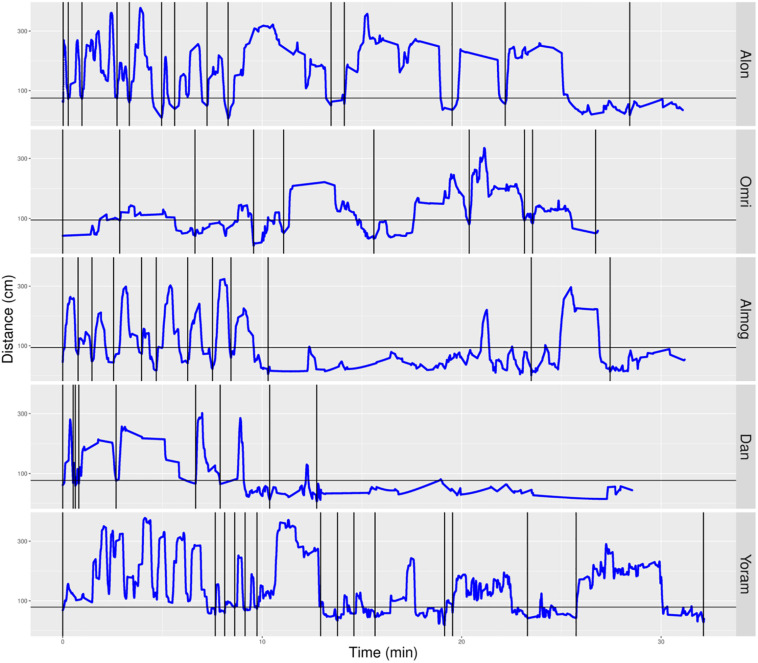
Distance from mother partitioned to excursions of typically developing (TD) infants. A plot of the TD infants’ management of distance from mother highlights the partitioning of the exploratory path into multiple modules, the extent of proximity between the centers of mass of the infant and the mother (sections below the horizontal line imply climbing on mother), and a tendency to start the session with sharp peaks (short durations of staying at the far end of excursions) and continue with flat-topped peaks (extended durations of staying at the far end of excursions. Once mother frees the infant, at the session’s onset, all infants except Omri move immediately away. Blue line plots distance from mother, black horizontal line marks mother’s customized boundary, and black vertical lines segment the plot into modular excursions.

**FIGURE 6:**
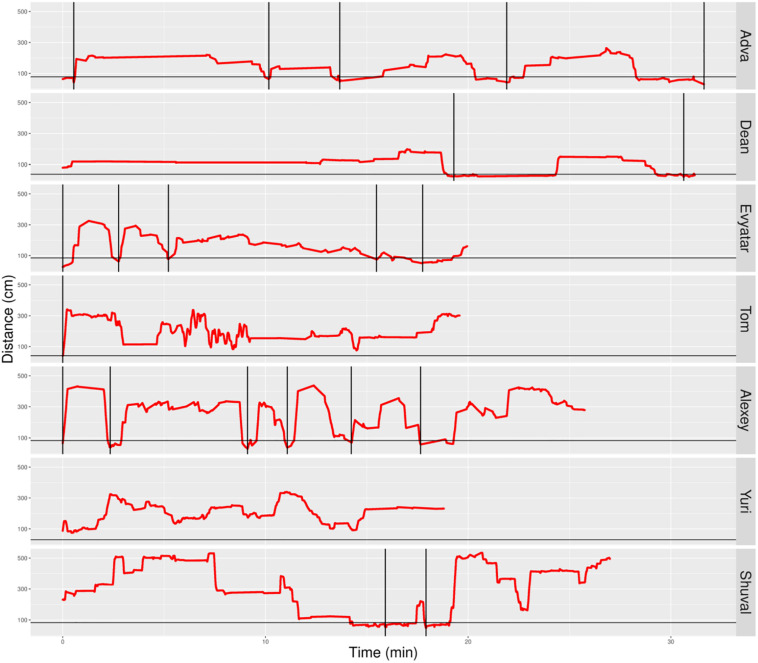
Distance from mother partitioned to excursions of typically developing (TD) infants. A plot of the non-typically developing (NTDs)’ management of distance from mother highlights few if any visits to mother, low proximity to mother (hardly any sections below the horizontal line), and a tendency to start the session with flat-topped peaks (long durations of staying at a specific distance, indicating long staying in place episodes). At the session’s onset, once mother frees them, all infants move away immediately. Only three of the infants end the session on mother’s lap. Red line plots distance from mother, black horizontal line marks mother’s customized boundary, and black vertical lines segment the plot into modules.

We parsed the infant’s path into excursions by using the zero crossing of the infant’s path with the horizontal line marking the boundary occupied by mother’s customized place (Figures 5, 6). Thereby, touching, or crossing the line on the way down and on the way up defines a visit to mother. A segment of the path located above the horizontal line and bounded by two zero crossings defines an excursion. As demonstrated in Figure 5, the most noticeable feature of the TD’s exploratory path is its partitioning into excursions. Four of the TDs (Alon, Almog, Dan, and Yoram) started the session with short duration excursions (marked by sharp peaks) and then proceeded to excursions that involved extended lingering episodes (marked by flat-topped peaks).

In contrast, the NTDs (Figure 6) performed very few if any excursions from mother: two excursions per session versus the median of 12 for the TDs. After adjusting for the different duration of the sessions the values were 0.397 and 0.118 excursions/min for the TDs and NTDs respectively, with effect size 0.33, and statistically significant (*p*-value 0.00253, Wilcoxon rank sum test).

The NTDs plots are characterized by long straight lines that maintain a relatively fixed distance in reference to the mother’s location, indicating that these infants are walking away from mother and staying away for long durations; whereas, the TDs plots are more dynamic and bounce back and forth across the line marking mother’s customized boundary (see section “Endpoint Summaries” – Average speed outside of mother’s vicinity). It should also be noted that the maximal distance from mother is much higher in the TDs, except for Shuval whose mother is located, unlike all the other mothers, at the opposite end of the room (see Figure 3). Unlike the TDs, who tend to terminate the session by climbing into mother’s lap, four NTDs terminate the session by the infant crying eliciting retrieval by mother.

### Physical Contact With Mother

**FIGURE 7:**
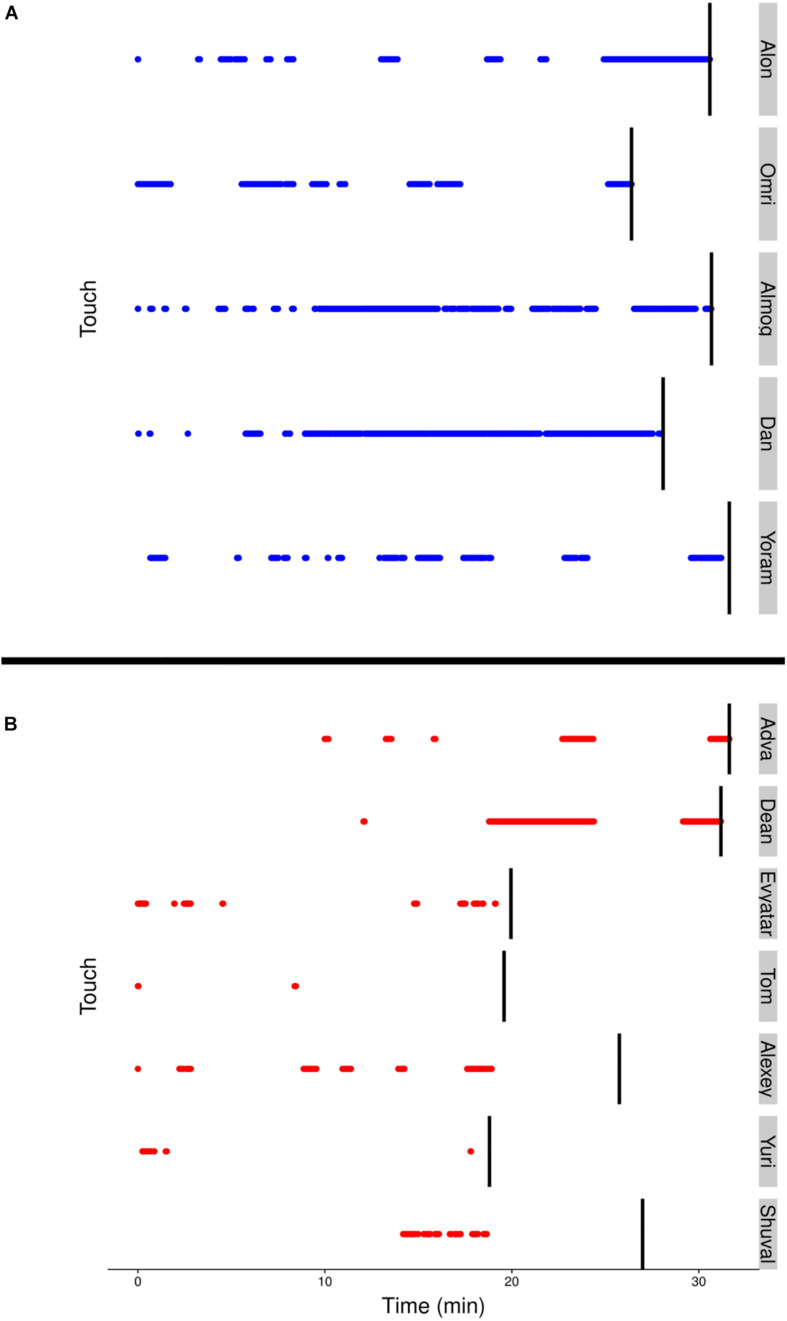
**(A)** The blue (TD) and **(B)** red (NTD) dotted lines, represent episodes of infants’ physical contact with mother. Management of physical contact with mother. The duration and frequency of episodes involving physical contact with the mother were high in the typically developing (TDs) and low or almost absent in the non-typically developing (NTDs). The vertical black lines represent the end of the respective infant’s session.

Although visits to mother do not necessarily imply physical contact, a major difference between the TDs and NTDs is the amount of physical contact they established with their mother (see section “Endpoint Summaries” – contact episodes per minute and proportion of contact time). All the TDs end the session with a relatively long contact episode (see Figure 7). In the NTDs only two infants end the session that way. Some of the short physical contact episodes were initiated by the mothers, who leaned forward and established physical contact with their nearby passing infant.

### The Itinerary, Duration, and Extent of Physical Proximity of Centers of Mass Between the Infant and Furniture Items During Visits to Furniture in the Room

The TDs visited the furniture items frequently and persistently, invading their respective places deeply and for long durations; whereas, the NTDs’ visits to the furniture tended to be infrequent and shallow (For elaboration of this aspect see Supplementary Figure S2 and accompanying text in Supplementary Material).

### Endpoint Summaries

**FIGURE 8:**
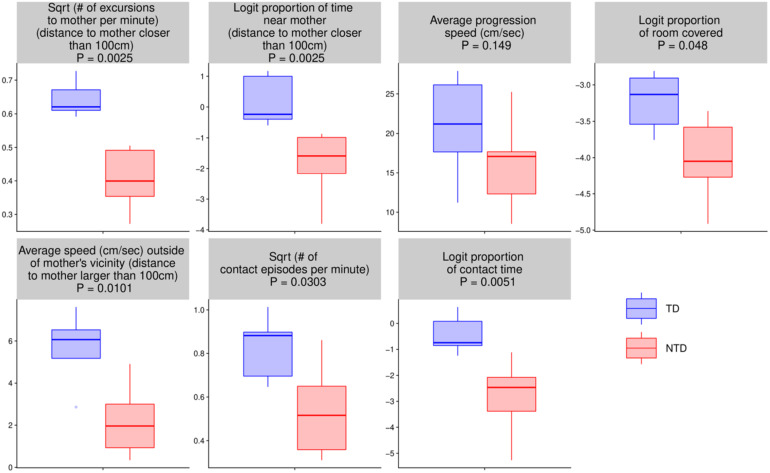
Endpoint summaries. Boxplot summaries demonstrate significant differences between the typically developing (TDs) and non-typically developing (NTDs) behavioral endpoints. All comparisons were conducted using Wilcoxon rank sum test. Except for average progression speed and logit of proportion of room covered, in all endpoints the differences between the TDs and NTDs are statistically significant (at significance level 0.05) after Benjamini–Hochberg (BH) adjustment for multiple comparisons (Benjamini and Hochberg, 1995).

For the seven quantitative endpoints summarized per infant, their values for one group versus the other are visualized by boxplots in Figure 8, and the differences are summarized numerically in Table 1. It can be seen that the differences between the TD and the NTD are consistent and large in all endpoints with effect sizes bigger than one, except for the average progression speed where even there it is 0.8. In spite of the small number of infants in each group, the differences for (i) the number of excursions from mother per minute, (ii) proportion of time near mother (iii) proportion of contact time with mother (iv) number of contact with mother episodes (v) and average speed outside mother’s vicinity, and (vi) proportion of contact time except for average progression speed and logit of proportion of room covered, the differences between the TDs and NTDs are statistically significant (at level 0.05, Wilcoxon rank sum test) and remain so after BH adjustment for the multiple comparisons (Benjamini and Hochberg, 1995). Moreover, for the number of excursions from mother per minute and for the proportion of time near mother there is complete separation between the TD and the NTD groups.

We also study the effect of age as a covariate, using regression analyses. The effect of age is not statistically significant in any of the regressions (with *p*-values 0.15–0.85), and the membership in the TD or NTD groups have age-adjusted effects of the same direction and similar in size to the unadjusted differences (see Supplementary Table S3).

### Excursions Versus One Un-Partitioned Path

The animations presented below demonstrate the partitioning of the path into excursions in two selected TDs:

Animation: Partitioning of Almog’s (TD) path into excursions (Supplementary Movie S3).

Animation: Partitioning of Alon’s (TD) path into excursions (Supplementary Movie S1).

Animations of partitioning of the paths of all the TDs are presented in the Supplementary Material [Alon (Supplementary Movie S1), Omri (Supplementary Movie S2), Almog (Supplementary Movie S3), Dan (Supplementary Movie S4), Yoram (Supplementary Movie S6)]. The TDs exhibit a highly mother-centered organization involving modular partitioning into excursions.

The animations of the paths of two selected NTDs presented below illustrate how the NTD paths tend to avoid mother’s place.

Animation: Partitioning of Yuri’s (NTD) path into excursions (Supplementary Movie S11).

Animation: Partitioning of Tom’s (NTD) path into excursions (Supplementary Movie S10).

Animations of half-hour, un-partitioned paths of all the NTDs are presented in the Supplementary Material [(Alexey (Supplementary Movie S5), Adva (Supplementary Movie S7), Dean (Supplementary Movie S8), Evyatar (Supplementary Movie S9), Tom (Supplementary Movie S10), Yuri (Supplementary Movie S11), Shuval (Supplementary Movie S12)].

## Discussion

### Mother Related Exploration

Mother-related exploration has been previously described in human infants in several studies: reinforcing Bowlby’s attachment theory with ethological data, Ainsworth observed that human infants use mother as a secure base for exploration (Ainsworth and Bowlby, 1991). Similarly, Mahler’s psychoanalytic separation-individuation theory on the psychological development of the human infant is largely supported by observations of the infants using mother as reference and “home base” (Mahler et al., 1975). Human infant performance of increasingly longer exploratory excursions has also been reported (Rheingold and Eckerman, 1970), and plotted (Vitelson, 2005), previously. The majority of these studies aimed at elucidating intrapsychic processes, such as symbiosis (Mahler et al., 1975), separateness, identity (Stern, 2009), self, object relationships, attachment, security, or separation anxiety (Ainsworth, 1969), on a day-by-day or month-by-month developmental scale (Rheingold and Eckerman, 1970; Jones, 1972) using verbal reports of behavior (Mahler et al., 1975). The current study complements these reports by introducing a phenotyping approach, using continuous kinematic observables for obtaining a comparative, structural, phylogenetic perspective on human infant exploration.

### The Two Basic Elements of Our Approach

Leaving the infants to their own devices had a profound effect on their behavior. Finding themselves in a novel, relatively pleasant environment, without being continuously bombarded by social stimuli, yet under the relatively silent visual attention of mother, the infants were entrusted with the full management, at their own pace and for an extended period of time, of their own location, distance, opposition (Eshkol and Wachman, 1958; Yaniv and Golani, 1987) contact, and extent of physical proximity with mother, furniture, and toys. In this way they disclosed to the observer through their behavior the endogenous constraints that shaped their attention, perception, and engagement with the physical and parental environment (Supplementary Figure S2A,B). By (i) mildly reducing the mother’s retrieval response in a safe environment and (ii) quantifying kinematics in a natural frame we uncovered commonalities as well as disparities between the TDs and the NTDs.

### The Hypotheses Supported by This Study

These two elements provide the computational and statistical evidence that support the hypotheses presented in the introduction: (1a) in a situation involving a mother and an infant where the mother attends passively to the infant from a stationary location, the exploratory behavior of pre-walking TD human infants is partitioned into natural modules called excursions, performed in reference to mother. (1b) The demonstrated architecture of TD human infant exploration, being composed of lingering episodes, progression segments, and origin-related excursions appears to be homologous to animal origin-related exploration, indicating the potential translational value of this behavior. (2a) This modularity is weak or even absent in NTDs, who came for assessment and treatment to a center for the early treatment of autism in infancy and (2b) these differences can be quantified by measuring properties of the paths performed by the infants in the two respective groups. Weak modularity can thus (2c) be used as an early warning signal.

### Exposing the Structure of TD Infants’ Behavior

Making no prior assumptions regarding mother-infant relationship, we first established each infant’s most preferred place in the environment and required that dwell-time would be incrementally accumulated in that place through the sequence of multiple visits. Having established the preference for mother’s proximity in the TDs, we found that the TDs paid a higher number of visits to mother. Furthermore, the TDs came to extensive grips with mother, not only by physically touching her during visits (Figure 7) but also by climbing into her lap as indicated by their distance shrinking to 0 (Figures 4–6). The parallel between the extensive and deep engagement of the TDs with mother and with the furniture items, versus the infrequent and shallow engagement of the NTDs with mother and with the furniture items is worth noting (see Supplementary Figure S2 and accompanying text in Supplementary Material).

Visits to mother were then used to partition the overall path into excursions that started and ended in mother’s vicinity (vertical bars in Figures 5, 6). Some of the TDs, like Almog, performed simple excursions with monotonical outbound and inbound portions. Others, like Alon also performed complex excursions that included several back-and-forth shuttles (Fonio et al., 2009) on the inbound portion of the excursion.

### The Homologous Primitives and Modules of Origin-Related Exploration

In comparative developmental anatomy, homologies are defined as modules sharing the same architectural plan [the same connectedness between primitives according to Saint-Hilaire’s (1822) principle of connections, and the same ancestral origin (Wagner, 2018)]. In the absence of behavioral fossils, homologies must, however, be defined in behavior, for the time being, as modules sharing the same connectedness (Golani, 1992; Gomez-Marin et al., 2016). In vertebrates, partitioning of exploratory behavior into excursions going away from an origin and returning to it is almost universal. The origin can be a home site in wild animals (Brown, 1966; Fritts and Mech, 1981; Hoffmann, 1984; Martin and Rudiger, 1988; Woodgate et al., 2016), a home base in adult laboratory rodents (Eilam and Golani, 1989; Wexler et al., 2018), a huddle of siblings in infant laboratory rats (Loewen et al., 2005), a place near the entrance door of an equestrian arena, where horses look out, pace and roll (Burke and Whishaw, 2020), or a mother in primates including human infants. Human TD infant exploration is thus first and foremost homologous to origin-related exploration across the vertebrates, because its architecture is the same as that of origin related behavior in vertebrates: in all the vertebrate taxa the primitives are lingering (staying-in-place) episodes and progression segments assembled into origin-related excursions, origin being defined as the place marked by highest cumulative time and highest number of visits (Golani and Benjamini, 2018). In addition, human TD infant exploration is homologous to primate mother-related exploration because in both primates and TD human infants mother’s location is used as the origin (van de Rijt-Plooij and Plooij, 1987). Our findings thus support the use of both origin and mother-related exploration as translational models.

The current study of homology is of explorative nature, merely portraying the contours of our future work-plan frame. To further support a homology between origin-related exploration in vertebrates, the excursion path should be parametrized in terms of further kinematic degrees of freedom that shape it – translation velocity, path curvature, and trunk orientation (Gomez-Marin et al., 2016). The excursion should also be segmented into progression segments and lingering episodes (Drai et al., 2000). It is also necessary to describe the relations among all the rigid moving parts of the kinematic linkage (Eshkol and Harries, 1998; Golani, 2012), including a continuous record of the infant’ and mother’ facing direction, recording their visual attention continuously (Sorce and Emde, 1981). The technology for this endeavor has just become available (Mathis et al., 2018).

### The Deficit in Path Modularity in the NTD Infants

The NTDs exhibited differences from the TDs in structure and quantity of visits to mother. The places of high dwell time and high frequency of visits were in mother’ proximity in all TDs but in none of the NTDs (Figures 2, 3). Moreover, in contrast to TD mothers, most of the NTDs’ mothers were visited rarely if at all (see boxplot summaries Figure 8). The segmentation of the path into excursions revealed that the TDs performed a median of 12 excursions per session whereas the NTDs performed 2 excursions per session, or comparing in excursions/minutes 0.397 vs. 0.118 excursions/minutes; three of the NTDs did not perform any excursions at all.

In the absence of a preferred stable place, the NTDs paths could not be segmented into excursions: they were punctuated like those of the TDs’paths by lingering episodes, but these episodes tended to be situated away from mother. As soon as the session started, both TDs and NTDs slid down from mother’s lap and crawled away, but while the TDs tended to immediately progress back and forth in reference to mother, lingering briefly in several locations along the excursion’s path, the NTDs performed an extended staying-in-place episode as soon as they reached a piece of furniture or a toy (Adva, Tom, Evyater, and Shuval), or as they stopped in an empty space away from walls (Dean), and tended to stay in place at that location for extended durations (Figure 6). Such extended staying-in-place episodes are also performed by TDs, but typically much later in the session, only after performing a sequence of short staying-in-place episodes (Figure 5 Alon, Almog, Dan, and Yoram). Importantly, the NTDs did not exhibit origin-related exploration in reference to any place or object in the room (e.g., Tom and Yuri).

### Early Signs of Autism?

The findings in the current study could lead us to associate the described behavior of the NTDs with early signs of autism, as these infants were referred to the Mifne Center for Early Treatment of Autism for assessment and/or treatment. We also were able to contact the parents of six of the seven NTDs: four of the six are in special education schools, and two of them are in mainstream schools following 1 and 2 years of intensive therapy. However, such a conclusion is beyond the capacity and confines of this study which is of exploratory nature. In order to make such an association it would be necessary to replicate (Kafkafi et al., 2017) the study with much larger groups of TDs and NTDs, concurrently performing follow-up developmental tests at older ages, and screening for developmental disorders, including ASD. The tracking and endpoint extraction should be completely automated and statistical analysis should be blinded from the group identity, with a clear pre-registered protocol. It may be also beneficial to shorten the session to 20 min as over longer period some mothers tend to reach toward their infants. Despite these limitations, we have, strictly speaking, found a candidate referral tool for child development specialists examining young pre-walking infants. Once having proved the replicability of this prodrome, machine learning classification using the relevant features could be extracted from videos of infants taken in an unforeseen natural environment, alerting for ASD risk in minutes (Tariq et al., 2018, 2019).

### Current Absence of Animal Model Exhibiting a Deficit in Modularity

A main motive of the current study has been the comparative analysis of origin-related exploration in human infants and animal models. This implied the search for symptoms exhibited during exploration in mother’s presence by NTD human infants, and then a subsequent search for the same symptoms in, e.g., ASD mouse models free exploration within our experimental paradigm, consisting of a home cage connected by a doorway to a large arena allowing free exploration (Fonio et al., 2009). So far, however, we have not been able to identify a mouse model that would perform active investigation of the arena without referring back to its home cage, or while being relatively free of the attraction of its home cage.

The paucity or absence of excursions in the NTDs is remarkable in view of the presence of mother-related exploration in primates, the sharing of origin-related behavior in vertebrates and several arthropods, and our inability so far to reproduce a deficit in homing in an experimental setup allowing free exploration. This inability should, however, be reconciled with findings that do show deficits in homing (Ognibene et al., 2007) and in early communication deficits in rodent models of ASD (Scattoni et al., 2008).

### Structure-First and Function-First Paradigms Are Complementary

Our interest has been to study the structure of human infant exploratory behavior in the presence of mother, isolate its candidate homological constituent measures, compare them across the TDs and NTDs, and use the differences in our search for corresponding translational animal models. This aim prioritizes the morphogenetic study of structure (Raff, 2012; Gomez-Marin et al., 2016), and views the mother-infant situation as a basic, natural situation requiring structural characterization and understanding before the application of experimental perturbations, while temporarily suspending judgment about function.

Infant carrying has evolved several times in the Primates and, once evolved, it has been conserved (Ross, 2001). Whereas in Primates carrying behavior is largely managed by the infant’s active riding behavior, in humans, due to the evolution of precociality, carrying is exclusively managed by the mother, allowing the infant to alternate between periods of utterly passive indulgent behavior and periods of initiation and active management of exploration. Precociality and active exploration of the environment coexist in human infants due to a partial delay in the onset of active infant exploration.

We do not deny the functions attributed to origin (Golani and Benjamini, 2018) or mother-related behavior: in rodents – the management of novelty and arousal (Fonio et al., 2009); in primates – socialization (Hinde and Simpson, 1975), in bumble bees – foraging (Woodgate et al., 2016), and in human infants – attachment and security (Mahler et al., 1975; Bowlby, 2012; Ainsworth et al., 2015), as well as peragration (Hoch et al., 2018). These studies complement other studies involving the motivation to explore (Berlyne, 1960), skilled action (Bruner, 1973), the ecological link between action and perception (Gibson, 1988), as well as current studies relating perception, development of locomotion, and exploration (Gibson, 1988; Adolph and Berger, 2007; Soska et al., 2010; Franchak et al., 2011; Kretch et al., 2014; Kretch and Adolph, 2017). The fact that a controversy between structure-first and function-first paradigms prevails for almost two centuries (Appel, 1987) in the study of biological phenomena including behavior, implies that both paradigms can be useful, depending on one’s aims.

Indeed, most classical researchers of human infant mother-related exploration prioritize the analysis of its functional aspects. Other studies engage with psychotherapy (Stern, 2009), psychoanalytic theory (Mahler et al., 1975), or the study of an infant’s emotional life (Bowlby, 2012), currently also in correlation to neural maturation (Schore, 2015). This includes, for example, the study of attachment (Ainsworth and Bowlby, 1991; Solomon and George, 1999; Bowlby, 2012), detachment (Rheingold and Eckerman, 1970), separation-individuation in the infant’s intrapsychic life (Mahler et al., 1975), sense of infant’s self (Stern, 2009), and inter-subjectivity (Trevarthen, 1979). Given their aims, these studies tend to ignore spatiotemporal continuity.

On the one hand, the excursion could be viewed as a manner of interaction between infant and mother (Stern, 2009), or even a joint attention (Moore and Dunham, 1995) affair managed mostly by the infant. For Mahler the “checking back pattern” part of the excursion is the most important fairly regular sign of beginning somato-psychic differentiation, being the most important normal pattern of cognitive and emotional development. This checking back pattern is, evidently, also a critical part of the excursion from a survival vantage point. Throughout primate history this behavior was required for survival by infants who were carried by their mothers, using her as an origin and haven during forays into the environment. On the other hand, the absence of origin related behavior in reference to any other place in the environment in the NTDs might reflect a wider deficit in organizing exploratory behavior. Either way, the homological status of this behavior suggests that like any other homology it is very hard to eradicate (Alberch, 1989), giving hope that it can be reactivated in young infants not showing it.

## Data Availability Statement

The datasets generated in this study with accompanying code can be found in https://github.com/tfrostig/Infants-Analysis.

## Ethics Statement

The study, involving human participants, was reviewed and approved by Tel Aviv University Ethics Committee. Written informed consent to participate in this study was provided by the participants’ legal guardian/next of kin.

## Author Contributions

GS and HA collected the data and performed the experiment. TF, IG, and YB analyzed the data. TF developed the algorithms, processed the videos and created the visualizations. IG, YB, and HA conceived the project. All authors participated in writing the manuscript.

## Conflict of Interest

The authors declare that the research was conducted in the absence of any commercial or financial relationships that could be construed as a potential conflict of interest.

## Footnotes

1 https://github.com/tfrostig/RSEE
