# Supplementary material for: Exploration in the Presence of Mother in Typically and Non-typically Developing Pre-walking Human Infants
Source: Front Behav Neurosci. 2020 Nov 13;14:580972. doi: 10.3389/fnbeh.2020.580972 (PMC7691591; doi:10.3389/fnbeh.2020.580972)
Supplement: Supplementary file 1 [file Data_Sheet_1.pdf]

Supplementary Material

# Supplementary Material

## 1 Physical proximity of centers plots.

**Figure S**1. Illustration of a physical proximity of centers plot including annotation. The black wedge represents the % of time the child spent near mother; the concentric circles radii represent the distance of the infant from mother; and the length of the arc at the respective radius represents the % of time spent at that distance (or greater).

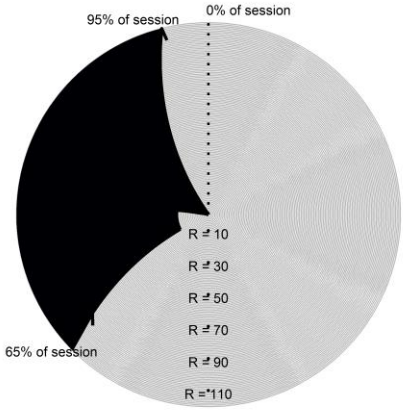


## 2 The itinerary, duration, and extent of physical proximity of centers of mass between the infant and furniture items during visits to furniture in the room.

Fig. S2 a, b presents plot summaries of the extent of proximity of each of the infants to mother, with the four items of furniture in the room, and with the two doorways leading out of the room: how each of the infants comes to grips with structures in the environment, and to what extent they come to grips with mother.

As in Figs. 4 a, b, starting at 12 o'clock and proceeding clockwise, all the visits paid by each of the infants to these items of furniture are plotted in their order of performance, including the relative start and end times, and the extent of physical proximity exhibited in each of the visits. Generally speaking, the TDs' proximity agendas were much more eventful and dynamic than those of the NTDs (evidenced by the number and variety of colored sections tiling the circles): multiple sharp spikes that touch the center in TDs (Almog, Dan, and Alon), versus a few extended sections of only a few colors that do not reach the center at all in NTDs (Adva, Dean, Evyater and Yuri). i) The TDs visited multiple items of furniture, paying multiple visits to each, whereas the NTDs visited the furniture much less frequently. ii) As evidenced by the extent of colored areas near the circles' centers, the TDs approached and often contacted the furniture, whereas the NTDs tended to approach the furniture less closely and invade it less deeply. iii) The large gray empty spaces in the NTD proximity graphs disclose the tendency of the NTDs to sometimes adhere to places that were distant from the furniture, either exhibiting stereotypies or investigating a toy, or perhaps attending to themselves rather than to the environment. Adva was exceptional in the NTD group in visiting mother at regular intervals, albeit only for five visits. Two infants, Alexey and Shuval, paid multiple visits to the doorway leading out of the room, trying to open it. The proximity graphs also highlight significant within-

group differences: e.g., while Almog alternated between visits to mother and single visits to an object across the session, Alon visited several items of furniture between successive visits to mother. The TDs took longer rests with mother, whereas the NTDs took longer rests near furniture.

**Figure S2**. The TDs' visited mother and the furniture items frequently and persistently, invading their respective places deeply and for long durations. **b** The NTDs' visits to mother and to the furniture were infrequent and shallow. For explanation of graphs see legend to Fig. 3.

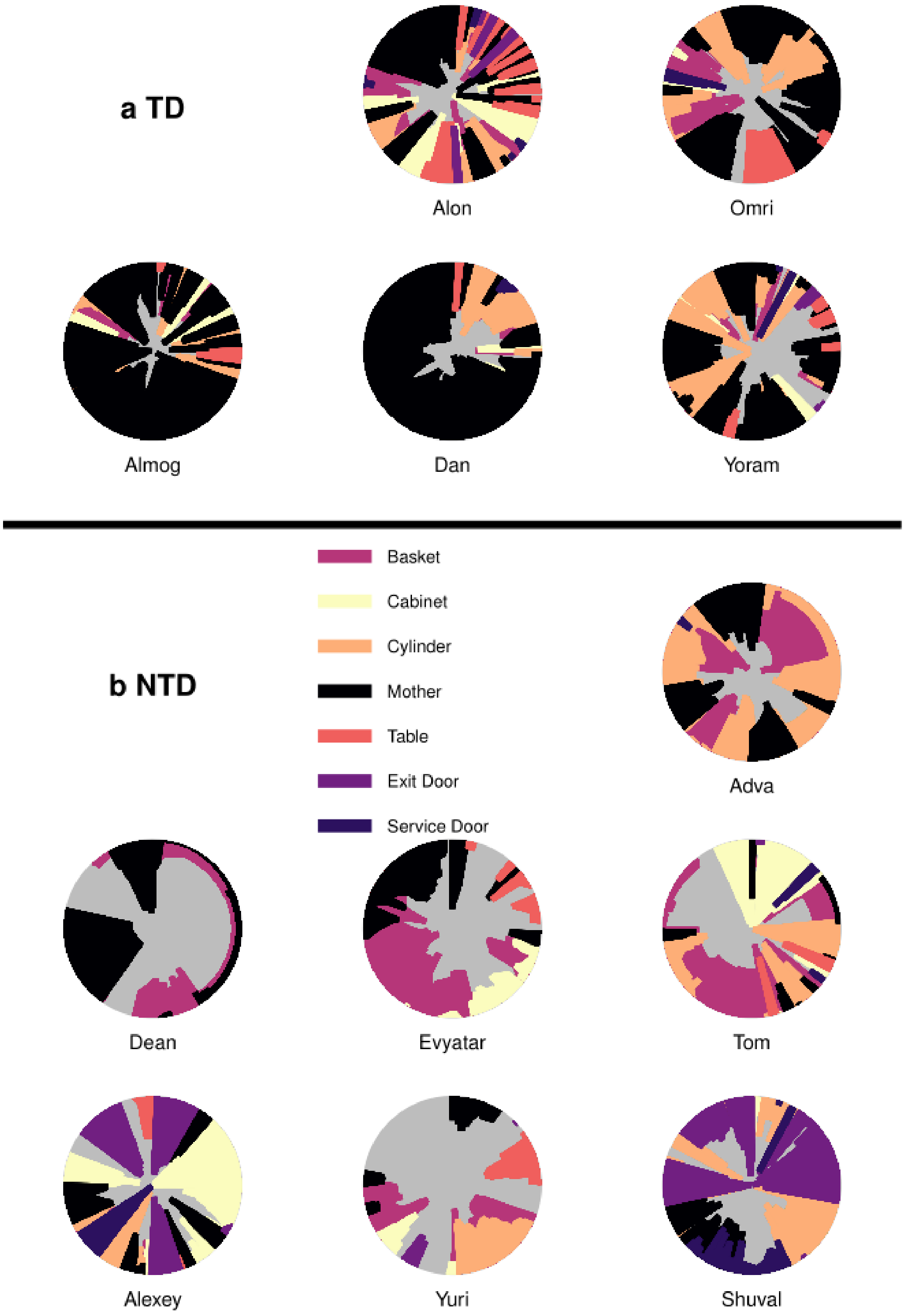

## 3 Quantitative examination of dwell time distribution in reference to mother.

To quantify dwell-time, we plotted the average time spent across the session for the whole range of distances from mother's center to up to 1.2m. The results reveal that the TD infants spent more time than the NTD infants within the entire range marking mother's vicinity. Using permutations to test the difference between the two average curves is significant (p-value 0.002, the average difference between the curves is 0.263).

**Fig S3 a, b, c, d**. Proportion of time spent in mother's vicinity. **c, d**: Number of visits to mother as a function of distance from mother. The CI for Figs. **b, d** was obtained using the normal approximation.

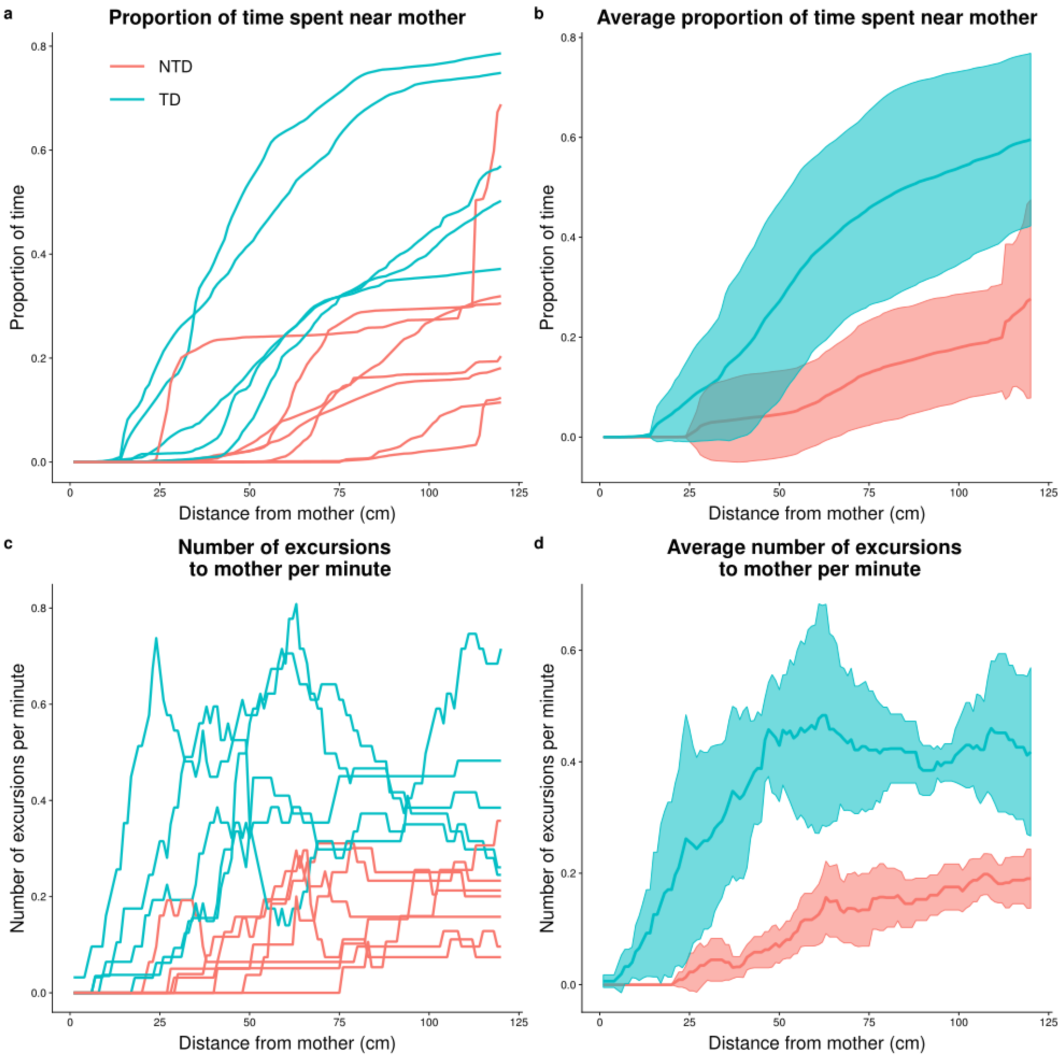


## 4 Quantitative summary of number of visits-to-places distribution in reference to mother.

Plotting the number of visits paid by each of the infants to the mother as we dynamically changed the inner radius of the two circles algorithm (the outer radius was kept at 1.1 of the inner radius), reveals that the number of visits to mother are higher for the TD compared to the NTD infants for the whole range of radii up to 1.2m. Since excursions are punctuated by visits to mother (see section *The infants' management of distance from mother*), it also follows that for all considered distances from

mother the TD infants exhibit more excursions than the NTD infants. Using permutations to test the difference between the average excursion's curves of the TD and NTD is highly significant (p-value 0.002, the average difference between the curves is 0.26 visits per minute).

**Table S1.** Summary of permutation testing of mean curves for the proportion of time spent near mother as a function of the radius and the number of excursions per minute as a function of the radius.

| Test (Comparing mean curves - permutation) | P-value | Adjusted p-value (BH) | Absolute means Difference $\|\bar{x} - \bar{y}\|$ |
|---|---|---|---|
| Proportion of time spent near mother ~ radius | 0.001998 | 0.001998 | 0.2635365 |
| # Excursions to mother per minute ~ radius | 0.000999 | 0.001998 | 0.2601119 |

## 5 Videos.

Animations of the infants' sessions were created and can be viewed using the following links. Note that the infant sometimes appears to be beyond the boundary of the room, which is due to the tracking of the child and then transforming the image, so that when the infant is standing it appears that the tracker is beyond the boundary. The mother's size changes from video to video, due to the way in which each mother sat (some were almost supine).

The infant's center of mass in each frame is represented by a black circle; movement in the current excursion is segmented into lingering segments and progression segments which are represented by blue and red colors respectively, past excursions are shown in grey; the mother position is in green and the rest of the furniture items are in black. The videos below can also be found in FigShare, accessible through the main text of the manuscript.

*TD infants:* Alon (movie S1), Omri (movie S2), Almog (movie S3), Dan (movie S4), Yoram (movie S6).

*NTD infants:* Alexey (movie S5), Adva (movie S7), Dean (movie S8), Evyatar (movie S9), Tom (movie S10), Yuri (movie S11), Shuval (movie S12).

## 6 Information about the NTD infants.

Shuval: Diagnosis before arrival to Mifne Center at age 15 months: PDD NOS & Attachment Disorder according to DSM-IV, diagnosed by a pediatric neurologist at a hospital near Tel Aviv. Shuval was a serious and quiet baby, avoided eye contact, and had repetitive and obsessive playing patterns. He had difficulties in eating.

Currently, Shuval attends a special education pre- school framework.

Dean: Diagnosis before arrival to Mifne Center at age 17 months: ASD according to DSM-5 - diagnosed by a pediatric neurologist at his HMO and diagnosed with hypsarhythmia at a local Hospital. He exhibited delayed motor and global development as well as a neurophysiological disorder. He was a passive baby.

Currently, Dean attends a special education pre- school framework.

Yuri: Diagnosis before arrival to Mifne Center at age 11 months: Suspected ASD - diagnosed by a pediatric neurologist at a Tel Aviv Hospital. Another diagnosis of ASD was given a year later by a pediatrician, a psychologist, and a speech clinician at a major US Autism Center. He exhibited motor and cognitive developmental delays. He was hypotonic with pronounced repetitive movements. His clinical picture pointed to severe autism.

Currently, Yuri attends a special education pre- school framework.

Evyatar: Diagnosis before arrival to Mifne Center at age 8 months: Suspected ASD - was diagnosed by a pediatrician and OT at a hospital in Jerusalem. He exhibited developmental delay in motor skills, difficulties in self-regulation, eating and sleeping problems. He did not make eye contact.

Currently, Evyatar has improved following two years of intensive therapy and currently attends a mainstream nursery.

Adva: Diagnosis before arrival to Mifne Center at age 11 months: Suspected ASD - diagnosed by a pediatric neurologist and psychologist at a hospital near Tel Aviv. Adva exhibited lack of eye contact, and lack of communication skills typical for her age. She did not show interest in people and in her surroundings. Her older sister is Autistic.

Currently, Adva has improved following one year of intensive therapy and attends a mainstream nursery.

Tom: Diagnosis before arrival to Mifne Center at age 18 months: Suspected ASD - diagnosed by a pediatrician and OT at a hospital near Tel Aviv. Tom exhibited lack of eye contact and lack of communication skills typical for his age. He was not treated in Mifne Center.

Currently, Tom parents were accessed and refused to give any information on his current state.

Alexey: Diagnosis before arrival to Mifne Center at age 11 months: Communication and language disorder – evaluated by a neuropsychologist at HMO. Alexey, one of twins, arrived at Mifne center due to concerns of his parents regarding his development compared to his brother. Alexey exhibited poor eye contact, not interested in playing or in interaction with others.

Currently Alexey was diagnosed with a communication and language disorder by a pediatrician and speech clinician and attends a special communication kindergarten.

Both Alexey and his brother were observed, the brother's session was terminated shortly because he tried to climb the cabinet and fell, crying. In the preliminary version of this paper (available at BioRxiv doi: https://doi.org/10.1101/350736 ) Alexey mistakenly was considered to belong to the NTD group and analyzed accordingly. Once their parents were contacted for the final version, it

became clear that the parents came because of Alexey. This fact was corroborated in Mifne Center. Hence in the current version he was assigned to his relevant group.

As a result, the analyses are available for both assignments. In terms of significance the difference in proportion of room covered remained statistically significant (old p-value 0.0152, new p-value 0.048, and after BH adjustment 0.056), while average progression speed is no longer statistically significant (old p-value 0.0411, new p-value 0.149). In contrast, the difference in number of contact episodes became statistically significant (old p-value 0.0649, new p-value 0.0303 and after BH adjustment 0.0423).

## 7 Information about the TD infants:

The TD infants were recruited from the nearby villages; they were recorded at the pre-walking freely crawling stage, whose ages turned out to be between 9 months to 11 months.

## 8 Age distribution and age adjusted analysis

**Table S2.** Infants' age.

| **TD** | | **NTD** | |
|---|---|---|---|
| **Name** | **Age** | **Name** | **Age** |
| Alon | 10 months | Shuval | 15 months |
| Omri | 10 months | Dean | 17 months |
| Almog | 9 months | Yuri | 11 months |
| Dan | 9 months | Evyatar | 8 months |
| Yoram | 11 months | Adva | 11 months |
| | | Tom | 18 months |
| | | Alexey | 11 months |

Since infants were recruited based on their developmental stage (pre-walking yet freely crawling) they differed in the age. To adjust for age as a covariate, we used linear regression analysis where each transformed endpoint was a response variable and membership in the TD or NTD groups being an explanatory variable and age as a covariate.

The effect of age is not significant for any of the endpoints, from 0.15 to 0.85 The direction of the age-adjusted TD vs NTD effect is consistent with the direction of the unadjusted effects found in Table 1, and their sizes are comparable.

**Table S3.** Summary of linear regressions conducted for each of the endpoints. The effect size of the TD vs NTD variable is the measured effect of being a TD rather than NTD.

| | Variable | Estimate | SD | t-statistic | P-value (Linear regression) | P-value (Linear regression, adjusted (BH)) | P-value (Wilcoxon test adjusted (BH), Table 1) |
|---|---|---|---|---|---|---|---|
| Sqrt (# of excursions to mother per minute) (Infant's distance to mother closer than 100cm) | Age | -0.007 | 0.0088 | -0.7882 | 0.4508 | | |
| | TD vs NTD | 0.2121 | 0.0555 | 3.8236 | 0.0041 | 0.0185 | 0.0088 |
| Logit proportion of time near mother (Infant's distance to mother closer than 100cm) | Age | -0.1543 | 0.1006 | -1.5345 | 0.1593 | | |
| | TD vs NTD | 1.4908 | 0.6305 | 2.3646 | 0.0423 | 0.0671 | 0.0088 |
| Average progression speed | Age | -0.4456 | 0.6901 | -0.6457 | 0.5346 | | |
| | TD vs NTD | 3.5604 | 4.3268 | 0.8229 | 0.4318 | 0.4318 | 0.149 |
| Logit proportion of room covered | Age | 0.0109 | 0.0564 | 0.1927 | 0.8515 | | |
| | TD vs NTD | 0.8096 | 0.3539 | 2.2877 | 0.048 | 0.0671 | 0.056 |
| Average speed outside mother vicinity (Infant distance to mother larger than 100cm) | Age | 0.2028 | 0.1809 | 1.1208 | 0.2914 | | |
| | TD vs NTD | 4.1441 | 1.1343 | 3.6535 | 0.0053 | 0.0185 | 0.0177 |
| Sqrt (# of contact episodes per minute) | Age | -0.0258 | 0.0195 | -1.3231 | 0.2184 | | |
| | TD vs NTD | 0.2154 | 0.1224 | 1.7594 | 0.1124 | 0.1311 | 0.0424 |
| Logit proportion of contact time | Age | -0.127 | 0.126 | -1.0082 | 0.3397 | | |
| | TD vs NTD | 1.9911 | 0.7899 | 2.5206 | 0.0327 | 0.0671 | 0.0118 |
